# Analysis of Clinical Characteristics of Refractory Food Protein-Induced Allergic Proctocolitis

**DOI:** 10.3390/children12111494

**Published:** 2025-11-04

**Authors:** Juan Zhang, Hui Wu, Jun Li, Xun Liu, Xueying Shi, Hua Zhang, Zailing Li

**Affiliations:** 1 Department of Pediatrics, Peking University of Third Hospital, Beijing 100191, China; arbooo@bjmu.edu.cn (J.Z.); 1663187505@bjmu.edu.cn (H.W.); 2 Department of Gastroenterology, Peking University of Third Hospital, Beijing 100191, China; bysylijun@bjmu.edu.cn (J.L.); daleliuxun@bjmu.edu.cn (X.L.); 3 Department of Pathology, Peking University of Third Hospital, Beijing 100191, China; shixueying@bjmu.edu.cn; 4 Department of Clinical Epidemiology Research Center, Peking University of Third Hospital, Beijing 100191, China; zhanghua@bjmu.edu.cn

**Keywords:** corticosteroids, food allergy, growth retardation, hemoglobin, pediatric proctocolitis, refractory FPIAP

## Abstract

**Highlights:**

**What are the main findings?**

- Refractory food protein-induced allergic proctocolitis (FPIAP) is associated with prolonged symptoms, growth retardation, and dietary intolerance.
- Non-early-onset FPIAP cases showed significantly more growth issues, lower hemoglobin levels, and higher corticosteroid use than early-onset cases.

**What is the implication of the main finding?**

- Early identification of FPIAP onset may help reduce the risk of growth delays and the need for corticosteroids.
- Individualized treatment strategies are needed to improve long-term outcomes and food tolerance in children with refractory FPIAP.

**Abstract:**

**Background/Objectives**: Food protein-induced allergic proctocolitis (FPIAP) is a non-immunoglobulin-E-mediated allergic colitis. Most cases resolve after 1 year of age, but delayed resolution and growth retardation may occur in some refractory cases. We aimed to explore the clinical characteristics, treatment approaches, and outcomes of such pediatric patients. **Methods**: We retrospectively analyzed 35 patients with refractory FPIAP at our center between January 2015 and January 2025. Patients were categorized into early- and non-early-onset groups according to timing of symptom onset; various clinical data were collected and treatment regimens were monitored. **Results**: The proportion of patients with growth retardation was significantly higher in the non-early onset group than in the early-onset group (73.3% vs. 35.0%, *p* = 0.041), whereas hemoglobin levels were higher in the early-onset group (118.95 ± 11.26 g/L vs. 107.93 ± 14.61 g/L, *p* = 0.017).The proportion of corticosteroid use was significantly lower in the early-onset group (15.0% vs. 60.0%; *p* = 0.011). During follow-up, among 35 patients, 14 (40%) could not tolerate certain foods, including cow’s milk (100%), eggs (42.9%), and wheat (35.7%). **Conclusions**: Refractory FPIAP was protracted, with a higher incidence of growth retardation, lower hemoglobin levels, and higher corticosteroid use in the non-early onset group. The optimal treatment approach should be explored.

## 1. Introduction

Food protein-induced allergic proctocolitis (FPIAP) is a frequently encountered non-immunoglobulin E (IgE)-mediated gastrointestinal food allergy in infants, typically presenting with variable stool abnormalities, including mucus and visible blood, and inflammation localized to the distal colon as seen on colonoscopy [1]. According to the 2023 ESPGHAN guidelines for the diagnosis, management, and prevention of cow’s milk protein allergy, exclusively breastfed infants with mild FPIAP and no other allergic manifestations may be managed conservatively by monitoring symptoms during the initial month of rectal bleeding, without initiating dietary restrictions, as the condition is generally regarded as benign and self-limiting [2]. Nevertheless, the necessity of treatment for FPIAP remains a subject of debate [3,4]. Clinically, some breastfed infants exhibit persistent hematochezia despite maternal elimination diets, though such cases are rarely reported in the literature and deserve greater clinical recognition [5]. Furthermore, certain intractable cases may demonstrate delayed symptom resolution and impaired growth [6]. This underscores the absence of evidence-based protocols for managing infants with severe, persistent FPIAP unresponsive to maternal dietary avoidance or elemental formula therapy [7].

Currently, no expert consensus or guidelines are available for the treatment of refractory FPIAP in children [8]. The newly released 2024 ESPGHAN guidelines on cow’s milk protein allergy in breastfed children recommend an initial observation for 1 month. If no improvement occurs, further maternal dietary restrictions or a switch to a special milk formula should be considered [7]. However, in clinical practice, owing to maternal anxiety, many mothers limit their diet to 2–4 weeks. If ineffective, they are switched to an elemental or extensively hydrolyzed formula.

Colonoscopy with histological examination is a valuable diagnostic approach in patients with ongoing symptoms and poor response to standard care. Endoscopic findings may include mild colitis with patchy erythema and lymphonodular hyperplasia (LNH), particularly in the distal colon, while histopathology often reveals eosinophilic infiltration of the lamina propria and muscularis mucosa [9]. In this work, most patients had already switched to hypoallergenic formulas for 4 weeks without significant clinical improvement at the time of colonoscopy.

This study aims to review and analyze the clinical features of refractory FPIAP, provide evidence for early identification and explore effective therapeutic approaches.

## 2. Materials and Methods

### 2.1. Study Participants

We performed a retrospective study. We selected pediatric patients clinically diagnosed with FPIAP and admitted to our general pediatric ward between January 2015 and January 2025 in a single center. Patients were screened from the hospital databases, using the following search strings: “rectal bleeding”, “allergic colitis”, and “hematochezia”. The diagnostic criteria for FPIAP were based on the 2017 expert consensus on the diagnosis and management of food-allergy-related gastrointestinal diseases [9]. We only selected patients with FPIAP diagnosis confirmed with the following: the onset of symptoms was within the first 12 months of life; the main clinical manifestations included bloody stools with mucus. When the diagnosis is unclear, endoscopy should be performed. The endoscopic findings are nonspecific and may include erythema, erosion, edema, ulcers, and hyperemia around the colonic lymphoid follicles. Histological examination of colonic biopsies may show a small number of eosinophilic granulocytes infiltrating the tissue, with few crypt abscesses forming.

The inclusion criteria of refractory FPIAP were as follows: (1) disease onset age < 12 months; (2) breastfed infants with no improvement in bloody stools after maternal dietary avoidance and after 4 weeks of amino acid formula intake or formula-fed infants with no improvement after 4 weeks of amino acid formula intake; (3) complete endoscopic examination; and (4) clinical manifestations and history not meeting the criteria for food protein-induced enterocolitis. The exclusion criteria were as follows: (1) clinical diagnosis of very early-onset inflammatory bowel disease (VEO-IBD); (2) presence of immunodeficiency; (3) presence of severe cardiac, hepatic, or renal disease; and (4) incomplete clinical data. The participants were divided into two subgroups, based on the time of the trigger food causing FPIAP symptoms: the early-onset group (EOG), consisted of infants in whom symptom onset within the first 3 months of life, which period the gut microbiota was less mature, whereas those with onset after 4 months comprised the non-early-onset group (nEOG). Growth retardation was defined as a decline of one major percentile in a child’s weight curve on the Chinese child growth curve chart after the onset of illness.

The study was conducted according to the guidelines of the Declaration of Helsinki and approved by the Peking University Third Hospital Medical Science Research Ethics Committees (approval number: [2025] Medical Ethics Review No. [283-01]; approval date: 2 April 2025).

### 2.2. Data Collection

We collected patient data, including demographic information (sex, age at onset, age at admission); clinical data (time of first bloody stool, feeding method, time of breast milk discontinuation, type and duration of special formula use before endoscopy); endoscopic findings; and pathological results. Moreover, we recorded hemoglobin levels, absolute eosinophil counts, cytomegalovirus (CMV) antibody levels, Epstein–Barr virus (EBV) antibody levels, CMV and EBV deoxyribonucleic acid (DNA) levels, tuberculin infection T-cell test results, routine stool tests with occult blood, stool tests for *Clostridium difficile* toxin and glutamate dehydrogenase antigen, and stool bacterial culture results. Additionally, we documented the medications administered to the patients and their outcomes.

### 2.3. Statistical Analysis

Quantitative data were subjected to normality test using the Shapiro–Wilk test. Normally distributed measurement data were expressed as means ± standard deviations (x ± s), and comparisons between the two groups were made using the two-sample *t*-test. Non-normally distributed measurement data were expressed as medians (interquartile ranges) [M (P25, P75)], and comparisons between the two groups were made using the Mann–Whitney U test. All nominal categorical variables were described as count and % relative frequency, and comparisons between the two groups were made using the Fisher’s exact probability test. *p*-values equal to or less than 0.05 indicated statistical significance. All statistical hypothesis tests were considered as two-sided. Statistical analyses were performed using SPSS version 26.0 (IBM Corp., Armonk, NY, USA).

## 3. Results

### 3.1. Patients’ Selection

Of 53 medical charts screened, excluded 18 cases, 5 cases were diagnosed with VEO-IBD, 5 cases were diagnosed with FPIES, 3 cases could not complete endoscopic examination, and 5 cases with missing follow-up information. A total of 35 cases fulfilled the diagnostic criteria for refractory FPIAP and were included.

### 3.2. Demographic Data

Thirty-five (25 male and 10 female) patients were included in this study. The age of symptom onset ranged from 1 to 9 months as shown in Table 1. The median age at onset was 3 (1, 6) months, and the median age at admission was 12 (9, 22) months.

### 3.3. Clinical Data

At symptom onset, 24 patients (68.6%) were breastfed, nine (25.7%) were mixed-fed, and two (5.7%) were formula-fed. The median duration of special formula use before admission was 5 (2, 12) months. The majority of the infants were switched to amino acid formula due to the persistent symptoms (Table 1).

**Table 1:** Comparison of Demographic and Clinical Characteristics of Patients.

Indicator	Value	Indicator	Value
Gender [No. (%)] Male 0–3 months 4–6 months 7–9 months Female	25 (71.4%) 14 (70.0%) 5 (62.5%) 6 (85.7%) 10 (28.6%)	Select Amino acid formula proportion Breastfeeding Mixed feeding Formula feeding	23/24 (95.8%) 8/9 (88.9%) 2/2 (100%)
Age distribution of symptom onset [No. (%)] 0–3 months 4–6 months 7–9 months	20 (57.1%) 8 (22.9%) 7 (20%)	Symptoms occurrence [No. (%)] Before complementary food introduction After complementary food introduction	30 (85.7%) 5 (14.3%)
Age of symptom onset [M (P25, P75), months]	3 (1, 6)	Family history of allergic diseases [No. (%)]	11 (31.4%)
Hospitalization age [M (P25, P75), months]	12 (9, 22)	Complementary food introduction time [M (P25, P75), months]	6 (6, 8)

No difference in sex distribution was observed between the two groups (*p* = 1.000). The median age at admission was 13.5 (12.0,24.75) and 12.0 (9.0,20.5) months in the EOG and nEOG, respectively, with no significant difference observed between the two groups (Z = −1.173, *p* = 0.241). At symptom onset, there was no difference in feeding methods between the two groups. Among the 35 infants, 30 (85.7%) exhibited clinical symptoms before the introduction of complementary foods, while five (14.3%) exhibited symptoms after complementary food introduction. At admission, seven infants did not receive complementary food because of recurrent symptoms despite the use of special formulas. Among the 28 infants who received complementary foods, the median age at introduction was 6 (6–8) months.

As shown in Table 2, the proportion of antibiotic use was higher in the nEOG than in the EOG (*p* = 0.019). The proportion of infants with a family history of allergic diseases was higher in the EOG than in the nEOG, but the difference was not statistically significant (*p* = 0.069). No differences in clinical symptoms, such as refusal to feed, reflux, eczema, diarrhea, stools with excessive mucus, bloody stools, and night crying, were observed between the two groups. The proportion of patients with growth retardation was significantly higher in the nEOG (*p* = 0.041). No significant difference in the proportion of malnutrition was observed between the two groups.

### 3.4. Laboratory Tests

All patients tested positive for fecal occult blood. Table 3 shows that there is no significant difference in *C. difficile* toxins detection was found between the two groups. Two cases in the nEOG had positive IgM antibodies against CMV, but CMV DNA was negative and intestinal mucosa pathology did not indicate CMV infection. EBV and T-SPOT test results were negative. The EOG had higher venous hemoglobin levels (118.95 ± 11.26 g/L) than the nEOG (107.93± 14.61 g/L; *p* = 0.017). No significant differences were observed in absolute eosinophil counts, vitamin A levels, or fecal calprotectin levels. Serum food-specific IgE testing showed a total sensitization rate of 37.1% (13/35); with no significant difference between groups. Of the 35 patients included, 10 (28.6%) were sensitized to more than two food types; this subset was equally distributed between the two groups, with five cases in each.

Owing to early onset and a prolonged disease course, 15 EOG (75.0%) and 13 nEOG (86.7%) patients underwent whole-exome sequencing, which did not indicate immune deficiency or single-gene-related IBD. In the EOG, genetic testing was not performed in five patients: one was unable to tolerate milk, and four achieved tolerance. In the EOG, 2 patients did not undergo genetic testing; however, they had achieved food tolerance during the follow-up period.

### 3.5. Endoscopic Examination

All patients underwent colonoscopy. In the nEOG, two patients (13.3%) underwent two colonoscopies, and two patients (13.3%) underwent three colonoscopies, which was statistically significantly different from the EOG (*p* = 0.026) (Table 4).

**Figure 1:**
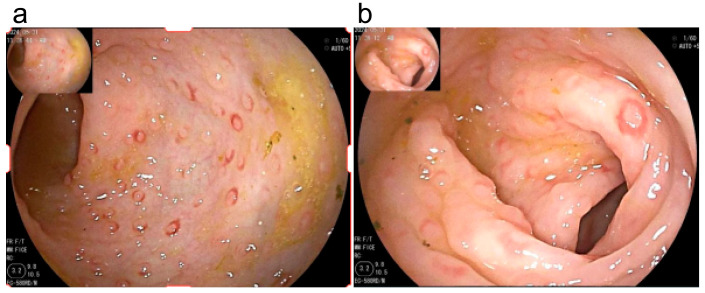
(**a**) Scattered multiple flat elevations in the sigmoid colon, presenting as LNH-like changes, with smooth mucosa, slightly red, and semi-transparent with circumferential congestion. (**b**) A flat elevation in the sigmoid colon with a small erosion on the surface.

The affected intestinal segments in both groups primarily extended from the ascending colon to the rectum, with more pronounced involvement of the left colon. Pathological changes were primarily characterized by LNH with erythema and erosion (Figure 1). Patients in the nEOG exhibited a broader extent of colonic involvement. Compared with the EOG, more cases of lymphonodular hyperplasia (LNH)-like changes with erythema were observed in the ascending, transverse, descending, and sigmoid colon, as well as the rectum; however, this difference was not statistically significant (Table 4). Pathology revealed mild to moderate mucosal inflammation in all cases, with some patients showing eosinophilic infiltration; however, the eosinophil count per high-power field did not meet the criteria defined in the guidelines for eosinophilic gastroenteritis [10].

Further, some patients underwent upper gastrointestinal endoscopy. In the EOG, 15 patients (75.0%) underwent one gastroscopy. In the nEOG, 11 patients (73.3%) underwent one gastroscopy, two (13.3%) underwent two gastroscopies, and two (13.3%) underwent three. Compared with the EOG group, the nEOG group exhibited significantly higher frequencies of erythema in both the gastric corpus and the antrum under endoscopy (Table 5).

### 3.6. Treatment and Outcomes

In the EOG, three patients were treated with predisone (0.5–1 mg/kg·d), and one of them received oral mesalamine. All three patients gradually tapered off the medication within 2 months, with improvement in clinical symptoms. In the nEOG, 9 patients were treated with predisone, two of whom used mesalamine (40–60 mg/kg·d); seven showed clinical improvement and tapered off within 2 months. Two patients showed no significant response to predisone; whom switched to infliximab treatment. Among the two patients treated with infliximab, one showed improvement after two infusions but was switched to oral thalidomide due to infusion reactions; another improved after two infusions and transitioned to oral thalidomide. The proportion of predisone use was significantly lower in the EOG (15.0%) compared to that in the nEOG (60.0%) (*p* = 0.011). And 83.3% (10/12) of patients use predisone showed efficacy.

During follow-up, 14 patients developed tolerance to foods in EOG, with a median time to tolerance of 24 (21.75, 37.25) months. In the nEOG, seven developed tolerance, with a median time to tolerance of 44 (24.00, 48.00) months. The proportion of tolerance in the EOG (70.0%) was not significantly different from that in the nEOG (46.7%) (*p* = 0.187).

In the EOG, six patients, and in the nEOG, eight patients—constituting 40% (14/35) overall—were unable to tolerate certain foods. The median follow-up age was 38.5 (21.75, 61.25) months. Among the intolerable foods, cow’s milk was the most common (100%), eggs (42.9%), wheat (35.7%), soybeans (21.4%), mutton (21.4%), seafood (14.3%), and nuts (14.3%).

## 4. Discussion

FPIAP is a non-IgE-mediated allergic colitis that typically presents in early infancy with bloody stools and increased stool mucus without significant malnutrition [1]. Recent literature reports a prevalence of 0.18% in healthy children and up to 64% in children with bloody stools [11,12,13]. FPIAP usually begins in the first few weeks of life, and most cases resolve after 1 year of age. The condition is characterized by distal colitis, with cow’s milk (65%) being the most common sensitizing agent, followed by eggs (19%), corn (6%), and soy and/or wheat (3%) [5]. Approximately 5% of infants have multiple food allergies [14].

Our study focused on infants with persistent symptoms despite changing an elemental formula for at least 4 weeks. We concluded that a familial predisposition to allergy may be associated with earlier onset of symptoms. Among the infants that received predisone, 83.3% (10/12) of patients showed efficacy, with most of them showing resolution after completing treatment.

Infants in the EOG had a higher proportion of family history of allergic diseases than those in the nEOG, which may suggest an association between familial predisposition to allergy and earlier onset of symptoms. However, this difference did not reach statistical significance, possibly due to the limitations of the single-center study design and small sample size. In particular, 85% of patients in the EOG had eczema, in consistency with the characteristics of the allergic march. The presence of atopic dermatitis has been identified as a risk factor for the development of FPIAP [15]. The proportion of antibiotic use was statistically significantly higher in the nEOG than in the EOG, suggesting that antibiotic administration in early life may increase an individual’s susceptibility to allergic diseases by altering the intestinal microbiota. Studies have found that antibiotic use within 6 months of age is associated with multiple food allergies. Antibiotics can exacerbate dysbiosis, disrupt the gut microbiota balance, and affect gut barrier function [16], potentially delaying the time to eventual food tolerance [17]. Although the literature reports a low proportion of FPIAP cases with malnutrition, this study found a rate of 17.5%, with no significant difference between the two groups. A study from Turkey reported that if FPIAP symptoms are protracted, dietary restrictions and a limited variety of complementary foods can lead to malnutrition [18]. Additionally, the nEOG had a significantly higher proportion of growth retardation, likely because these patients developed symptoms after 3 months of age. Owing to prolonged symptoms, long-term dietary restrictions, and limited nutrient intake, parents often stopped introducing new foods upon observing changes in stool characteristics and gastrointestinal symptoms, leading to a decline in the growth curve as the child aged.

FPIAP can present with bloody or occult blood in the stool. In this work, all infants had mucous and bloody stools with positive fecal occult blood test results. However, a positive fecal occult blood test alone is insufficient for diagnosing FPIAP because some normal infants may test positive [19]. When encountering infants with bloody or mucous stools, it is essential to differentiate FPIAP from intestinal infections, VEO-IBD, anal fissures, and polyps. In this study, no evidence of EBV or CMV infection was found in the intestinal mucosal pathology of the infants. Fecal calprotectin levels showed no significant differences between the groups, and no specific cutoff value currently exists for calprotectin in diagnosing FPIAP [20]. Hemoglobin levels were lower in the nEOG than in the EOG. A study conducted in the USA found that infants with FPIAP and persistent bloody stools are prone to iron deficiency and anemia [21]. Dysbiosis of early-life gut microbiota is associated with food allergies. In this study, four patients tested positive for *Clostridium difficile* toxin and 20 tested positive for glutamate dehydrogenase. Studies have found that an increase in *Clostridium difficile* at 3 weeks of age is associated with the development of food allergies in the first year of life [22]. While it remains unclear whether *Clostridium difficile* toxin positivity results from microbiota changes after allergies or whether microbiota changes predispose infants to allergies, infants are known to have a certain carriage rate of *Clostridium difficile*. One study found that *Clostridium perfringens* colonization is associated with the development of food allergies and cow’s milk protein allergy in Chinese infants [23], suggesting a potential link between gut microbiota dysbiosis and food allergies.

The nEOG underwent more colonoscopies than the EOG, likely due to the later onset of symptoms. Following the introduction of complementary foods, the symptoms recurred and did not fully resolve, necessitating repeat endoscopy to exclude other etiologies. Colonoscopic findings predominantly showed changes, such as erythema, erosion, and ulcers [24], with a higher proportion of ulcerative lesions observed in the ascending, descending, and sigmoid colon in the nEOG compared to the EOG. However, most of the patients had lesions concentrated in the left colon. Pathological changes were primarily LNH with erythema and erosion, in consistency with previous reports [25,26].

Most patients experienced symptom improvement after maternal dietary avoidance of allergens or switching to extensively hydrolyzed or elemental formulas. However, some refractory cases have been reported in clinical practice. Lake reported on 21 infants with persistent symptomatic proctitis lasting more than 6 months, who showed no significant clinical improvement after 4 weeks of hypoallergenic formula feeding. These infants continued to present with symptoms, such as mucous stools, bloody stools, or persistently positive fecal occult blood tests [21]. In another cohort study, seven (18%) children had cow’s milk protein allergy persisting beyond 1 year of age, and five infants demonstrated multiple food allergies. Other authors have indicated that risk factors for multiple food allergies include concurrent atopic dermatitis, high eosinophil levels at diagnosis, and sensitization to food allergens (skin prick test or specific IgE) [3,6]. In this study, 80% of infants in both groups had eczema, in consistency with previous reports. In both groups, 95% of infants received an amino acid formula for at least 4 weeks before admission but showed no significant clinical relief. For this special population, further evaluation by a pediatric gastroenterologist is required, including a nutritional assessment, endoscopic evaluation, and the exclusion of other potential diseases [18]. Additionally, attention should be given to the possibility of multiple food allergens, especially in the nEOG, where infants are in the stage of complementary food introduction. Multiple food allergies may cause recurrent symptoms during the introduction of complementary foods, leading to a prolonged disease course. In this study, 40% of the patients were unable to tolerate certain foods, with the most common foods being cow’s milk (100%), eggs (42.9%), wheat (35.7%), soybeans (21.4%), mutton (21.4%), seafood (14.3%), and nuts (14.3%). This is largely consistent with previous reports on the two most commonly intolerant foods [5].

Despite the clinical severity in these patients, common endoscopic findings include mucosal irregularities with LNH-like changes, often accompanied by erythema, surface erosion, and ulcers. Some patients exhibit mucosal erythema or erosive changes in the upper gastrointestinal tract. Consistent with previous reports, three out of 65 patients (4.61%) showed esophageal erythema on esophagogastroduodenoscopy (EGD), and 49 of 65 patients (75.38%) had eosinophilic infiltration on EGD biopsy [26].

A differential diagnosis is crucial for these patients, especially when the onset of symptoms in infancy requires differentiation from monogenic hereditary inflammatory bowel diseases. The identification of specific sensitizing foods remains a significant challenge. Currently, no reliable laboratory or skin tests are available to confirm non-IgE-mediated food allergies. A previous work discussed the use of food-specific IgG antibodies and skin patch tests, though their effectiveness requires further clinical and scientific validation [1].

Food allergies are characterized by type 2 inflammation. After exposure to food allergens and immune activation, dendritic cells transform Tregs into T Helper Type 2 (Th2) cells. The expansion of the Th2 cell population provides a local reservoir of interleukin (IL)-4, a key cytokine that sustains Th2 cell responses, promotes IgE switching in B cells, enhances mast cell survival, and increases tissue sensitivity to mast cell mediators [27]. Additionally, proliferating type 2 innate lymphoid cells can produce IL-4 and IL-13 [28]. Glucocorticoids, classic anti-inflammatory drugs, inhibit T cell activation, suppress the production of pro-inflammatory cytokines, and inhibit the chemotaxis of inflammatory cells in many type 2 inflammatory diseases. Therefore, this is a viable treatment option for patients with FPIAP. However, as most FPIAP cases have a favorable prognosis, they can be managed using a watch-and-wait approach. Glucocorticoids inhibit inflammation in refractory cases. In this study, three patients in the EOG and 9 patients in the nEOG were treated with oral prednisone, with 83.3% (10/12) achieving clinical remission within 2 months.

Tumor necrosis factor-alpha (TNF-α) is a key cytokine associated with chronic inflammatory diseases and has been shown to alter tight junctions between epithelial cells [29]. This suggests that it may play a role in the pathogenesis of FPIAP by altering the barrier function of intestinal epithelial cells. Based on this evidence, a study measured TNF-α concentrations in peripheral blood mononuclear cells from three groups of children and found that those with gastrointestinal food allergies (including allergic colitis) had significantly higher TNF-α concentrations compared to the control and IgE-mediated cow’s milk protein allergy groups [30]. Therefore, TNF-α may represent a potential therapeutic target. In this study, two infants in the nEOG, after excluding VEO-IBD and obtaining full informed consent from their parents, were treated with infliximab. The number of treatments was two, with symptom improvement observed. One patient discontinued treatment because of allergic reactions.

Mesalamine, a 5-aminosalicylic acid derivative, exhibits local anti-inflammatory activity in the gut and is commonly used to treat ulcerative colitis. This retrospective study evaluated the efficacy of mesalamine in infants with severe FPIAP. Among 65 infants (mean age, 2.98 ± 1.88 months), 44 with persistent rectal bleeding were treated with oral mesalamine. Twenty-one infants who used an amino acid formula and received mesalamine treatment for an average of 100 days showed significant improvement in reflux or vomiting, diarrhea, irritability, appetite, growth and development, and bloating. In the mesalamine treatment group, 81% of infants showed resolution of bloody stools, compared to 71.4% in the control group, with no significant difference between the groups. At 15 months after reintroducing cow’s milk, the recurrence proportion of bloody stool was lower in the mesalamine group (22%) than in that of the control group (85%) [26]. In this study, mesalamine was combined with other medications in three infants, suggesting its potential for treating FPIAP. However, the evidence level is low, and further clinical studies are needed to verify its efficacy and explore its mechanisms.

Follow-up of the cases revealed that the time to tolerance was longer than reported in many other studies, where most infants with FPIAP developed tolerance by the age of 1 year. In our study, infants in the EOG and the nEOG developed tolerance at 24 and 44 months, respectively. Previous studies have shown that infants with FPIAP whose initial symptoms include diarrhea tend to develop tolerance at a later age (approximately 30 months) [11], with 5% of infants not achieving tolerance until after the age of 3 years [31]. In the present study, 40% of infants remained unable to tolerate certain foods at a median age of 38.5 months (range: 21.75–61.25 months), a timeline that is later than the food tolerance ages reported in previous studies.

This study centered on children with refractory FPIAP. Currently, there is no clear definition for this patient group, and relevant research is limited. Differentiating it from diseases such as VEO-IBD is crucial for diagnosis. Yet, long-term follow-up revealed that some patients gradually tolerated foods with age, while others remained intolerant to certain foods. This indicates that food-allergic gastrointestinal diseases do not always lead to natural tolerance with age, and there is still no consensus on treating such patients. Given the small sample size of the current study, we cannot recommend the optimal treatment approach for such children with refractory FPIAP. Multi-center studies with a larger sample size are needed to clarify the clinical characteristics and optimal treatment approach.

## 5. Conclusions

Our study suggests that refractory FPIAP is more prevalent in clinical practice than previously recognized. Patients often recover slowly, even with food avoidance, and those with nEOG FPIAP exhibit a higher incidence of growth retardation and lower hemoglobin levels compared to those in EOG, primarily due to dietary restrictions and multiple food allergies. For refractory FPIAP, it is crucial to perform comprehensive endoscopic examinations, along with ongoing follow-up and guidance, to rule out VEO-IBD and other intestinal infections. Clinicians should remain vigilant in cases of persistent bloody or mucous stools, especially when eczema is present. Over time, some children may develop tolerance, while others may continue to be intolerant to one or more foods. Ensuring adequate nutritional support for these children’s growth and development is especially important.

Our findings may point to a new subtype of food-allergic gastrointestinal disease; however, its underlying mechanisms and markers remain unclear. With parental consent, anti-inflammatory treatments, such as mesalamine, predisone, thalidomide, and biologics, may be considered, although their efficacy needs further clinical validation.

## Figures and Tables

**Table 2:** Comparison of Medical History and Clinical Symptoms between the EOG and nEOG.

Symptom	EOG (n = 20)	nEOG (n = 15)	χ^2^ Value/Z	*p*-Value
Gender (Male) Hospitalization age [M (P25, P75), months] Feeding method [No. (%)] Breastfeeding Mixed feeding Formula feeding Special formula use time before hospitalization [M (P25, P75), months] Complementary food introduction time [M (P25, P75), months] Family history of allergies	14 (70.0%) 13.5 (12.0, 24.75) 14 (70.0%) 6 (30.0%) 0 (0%) 8.0 (4.25, 17.75) 6.0 (6.0, 8.0) 9 (45.0%)	11 (73.3%) 12.0 (9.0, 20.5) 10 (66.7%) 3 (20.0%) 2 (13.3%) 4.5 (1.25, 13.25) 6.5 (6.0, 7.75) 2 (13.3%)	- −1.173 ^b^ - −1.804 ^b^ −0.075 ^b^ -	1.000 ^a^ 0.241 0.314 ^a^ 0.071 0.940 0.069 ^a^
Antibiotic use	5 (25.0%)	10 (52.6%)	-	0.019 ^a^
Refusal to feed	1 (5.0%)	2 (13.3%)	-	0.565 ^a^
Reflux	2 (10.0%)	1 (6.7%)	-	1.000 ^a^
Eczema	17 (85.0%)	11 (73.3%)	-	0.43 ^a^
Diarrhea	18 (90.0%)	10 (66.7%)	-	0.112 ^a^
Stools with excessive mucus	18 (90.0%)	12(80.0%)	-	0.631 ^a^
Bloody stools	20 (100%)	12 (80.0%)	-	0.07 ^a^
Night crying	2 (10.0%)	2 (13.3%)	-	1.000 ^a^
Growth retardation	7 (35.0%)	11 (73.3%)	-	0.041 ^a^
Malnutrition	4 (20.0%)	3 (20.0%)	-	1.000 ^a^

^a^ Fisher’s exact probability test. ^b^ Z value. Abbreviations: EOG: Early-Onset Group, nEOG: Non-Early-Onset Group.

**Table 3:** Laboratory Test Results of the Two Groups of Children.

Laboratory Tests	EOG (n = 20)	nEOG (n = 15)	χ^2^/t/Z Value	*p*-Value
Positive for *Clostridium difficile* toxin	3 (15.0%)	1 (6.7%)	-	0.619 ^a^
Positive for glutamate dehydrogenase antigen	13 (65.0%)	7 (46.7%)	-	0.321 ^a^
CMV IgM antibody positivity	0 (0%)	2 (13.3%)	-	0.176 ^a^
Hemoglobin (g/L)	118.95 ± 11.26	107.93 ± 14.61	2.521 ^b^	0.017
Absolute eosinophil count [M (P25, P75), ×10^9^/L]	0.17 (0.060, 0.313)	0.23 (0.09, 0.33)	−0.634 ^c^	0.526
Vitamin A (μmol/L)	0.85 ± 0.34 (n = 12)	0.71 ± 0.21 (n = 8)	1.045 ^c^	0.310
Fecal calprotectin [M (P25, P75), μg/g]	116.96 (n = 11) (58.0, 263.98)	116.65 (n = 9) (42.51, 195.83)	−0.418 ^c^	0.676
Allergen-specific IgE sensitization rate	6/20 (30.0%)	7/15 (46.7%)	-	0.481 ^a^

^a^ Fisher’s exact probability test; ^b^ t value. ^c^ Z value. Abbreviations: EOG: Early-Onset Group, nEOG: Non-Early-Onset Group, CMV: Cytomegalovirus, Ig: Immunoglobulin.

**Table 4:** Colonoscopy Findings.

Item	EOG (n = 20)	nEOG (n = 15)	*p*-Value
Number of colonoscopies 1 time 2 times 3 times	20 (100%) 0 0	11 (73.3%) 2 (13.3%) 2 (13.3%)	0.026 ^a^
Involved intestinal segments Terminal ileum Ileocecal valve Ascending colon Transverse colon Descending colon Sigmoid colon Rectum	6 (30.0%) 3 (15.0%) 12 (60.0%) 13 (65.0%) 17 (85.0%) 18 (90.0%) 18 (90.0%)	5 (33.3%) 3 (20.0%) 13 (86.7%) 14 (93.3%) 15 (100%) 15 (100%) 15 (100%)	1.000 ^a^ 1.000 ^a^ 0.134 ^a^ 0.101 ^a^ 0.244 ^a^ 0.496 ^a^ 0.496 ^a^
Lesion characteristics in terminal ileum Uneven mucosa Erythema Erosion	5 (25.0%) 2 (10.0%) 1 (5.0%)	4 (26.7%) 3 (20.0%) 1 (6.7%)	1.000 ^a^ 0.631 ^a^ 1.000 ^a^
Lesion characteristics in ileocecal valve Erythema	3 (15.0%)	3 (20.0%)	1.000 ^a^
Lesion characteristics in ascending colon LNH with erythema Erosion Ulcer	12 (60.0%) 2 (10.0%) 0	13 (86.7%) 3 (20.0%) 2 (13.3%)	0.134 ^a^ 0.631 ^a^ 0.176 ^a^
Lesion characteristics in the transverse colon LNH with erythema Erosion Ulcer	13 (65.0%) 4 (20.0%) 1 (5.0%)	14 (93.3%) 3 (20.0%) 2 (13.3%)	0.101 ^a^ 1.000 ^a^ 0.565 ^a^
Lesion characteristics in the descending colon LNH with erythema Erosion Ulcer	17 (85.0%) 4 (20.0%) 0	15 (100.0%) 5 (33.3%) 1 (6.7%)	0.244 ^a^ 0.451 ^a^ 0.429 ^a^
Lesion characteristics in the sigmoid colon LNH with erythema Erosion Ulcer	17 (85.0%) 5 (25.0%) 0	15 (100.0%) 6 (40.0%) 1 (6.7%)	0.244 ^a^ 0.467 ^a^ 0.429 ^a^
Lesion characteristics in the rectum LNH with erythema Erosion Ulcer	18 (90.0%) 5 (25.0%) 1 (5.0%)	15 (100.0%) 5 (33.3%) 2 (13.3%)	0.496 ^a^ 0.712 ^a^ 0.565 ^a^
Eosinophil presence in pathology	4 (20.0%)	7 (46.7%)	0.144 ^a^

^a^ Fisher’s exact probability test. Abbreviations: EOG: Early-Onset Group, nEOG: Non-Early-Onset Group, LNH: Lymphonodular Hyperplasia.

**Table 5:** Gastroscopy Findings.

Item	EOG (n = 20)	nEOG (n = 15)	*p*-Value
Number of gastroscopies 0 1 time 2 times 3 times	5 (25.0%) 15 (75.0%) 0 0	1 (6.7%) 11 (73.3%) 1 (6.7%) 2 (13.3%)	0.085 ^a^
Involved sites and lesion characteristics Cardiac ulcer Fundic erythema Corpus erythema Angular incisura erythema Antral erythema Antral erosion Duodenal bulb erosion Erosion of the descending part of the duodenum	1 (6.7%) 1 (6.7%) 3 (20.0%) 0 (0.0%) 5 (33.3%) 3 (20.0%) 5 (33.3%) 2 (13.3%)	3 (21.4%) 6 (42.9%) 8 (57.1%) 4 (28.6%) 5 (35.7%) 3 (21.4%) 2 (14.3%) 3 (21.4%)	0.330 ^a^ 0.035 ^a^ 0.060 ^a^ 0.042 ^a^ 1.000 ^a^ 1.000 ^a^ 0.390 ^a^ 0.651 ^a^
Eosinophil presence in pathology	2 (13.3%)	2 (14.3%)	1.000 ^a^

^a^ Fisher’s exact probability test.

## Data Availability

Data will be made available on request due to ethical reasons. However, partial anonymized data can be requested from the corresponding author upon reasonable research requests, subject to the approval of the Ethics Committee and compliance with privacy protection requirements.

## Abbreviations

The following abbreviations are used in this manuscript:

CMV	Cytomegalovirus
DNA	Deoxyribonucleic Acid
EBV	Epstein–Barr Virus
EOG	Early-Onset Group
ESPGHAN	European Society for Paediatric Gastroenterology, Hepatology and Nutrition
FPIAP	Food Protein-Induced Allergic Proctocolitis
IgE	Immunoglobulin E
LNH	Lymphonodular Hyperplasia
nEOG	Non-Early-Onset Group
SPSS	Statistical Package for the Social Sciences
TNF-α	Tumor Necrosis Factor Alpha
T-SPOT	Tuberculin Infection T-cell Test
VEO-IBD	Very Early-Onset Inflammatory Bowel Disease

## References

[1] Vandenplas Y., Broekaert I., Domellöf M., Indrio F., Lapillonne A., Pienar C., Ribes-Koninckx C., Shamir R., Szajewska H., Thapar N. (2024). An ESPGHAN position paper on the diagnosis, management, and prevention of Cow’s milk allergy. J. Pediatr. Gastroenterol. Nutr..

[2] Barni S., Pessina B., Fioretti L., Scarallo L., Di Siena A., Bramuzzo M., Liccioli G., Sarti L., Tomei L., Giovannini M. (2025). Food Protein-Induced Allergic Proctocolitis: Real-World Experience from an Italian Cohort. Nutrients.

[3] Uncuoğlu A., Aydoğan M., Şimşek I.E., Çöğürlü M.T., Uçak K., Acar H.C. (2022). A prospective assessment of clinical characteristics and responses to dietary elimination in food protein-induced allergic proctocolitis. J. Allergy Clin. Immunol. Pract..

[4] Vandenplas Y., Meyer R., Hoffman I., Alliet P., De Greef E., Hauser B., Huysentruyt K. (2025). Discrepancies in the Diagnosis, Treatment and Prevention of Cow Milk Allergy Among Paediatricians. Acta Paediatr..

[5] Mennini M., Fiocchi A.G., Cafarotti A., Montesano M., Mauro A., Villa M.P., Di Nardo G. (2020). Food protein-induced allergic proctocolitis in infants: Literature review and proposal of a management protocol. World Allergy Organ. J..

[6] Cetinkaya P.G., Kahveci M., Karaatmaca B., Esenboga S., Sahiner U.M., Sekerel B.E., Soyer O. (2020). Predictors for late tolerance development in food protein-induced allergic proctocolitis. Allergy Asthma Proc..

[7] Salvatore S., Folegatti A., Ferrigno C., Pensabene L., Agosti M., D’Auria E. (2024). To diet or not to diet: This is the question in food-protein-induced allergic proctocolitis (FPIAP)-a comprehensive review of current recommendations. Nutrients.

[8] Mennini M., Felici E., Di Nardo G. (2025). The need for standardization in the diagnosis and management of food protein-induced allergic proctocolitis (FPIAP): The time has come to act. Minerva Pediatr..

[9] Li Z., Gong S. (2017). The Subspecialty Group of Gastroenterology, the Society of Pediatrics, Chinese Medical Association. Expert consensus of food allergic gastrointestinal disease. Zhonghua Er Ke Za Zhi.

[10] Papadopoulou A., Amil-Dias J., Auth M.K.H., Chehade M., Collins M.H., Gupta S.K., Gutiérrez-Junquera C., Orel R., Vieira M.C., Zevit N. (2024). Joint ESPGHAN/NASPGHAN guidelines on childhood eosinophilic gastrointestinal disorders beyond eosinophilic esophagitis. J. Pediatr. Gastroenterol. Nutr..

[11] Senocak N., Ertugrul A., Ozmen S., Bostanci I. (2022). Clinical features and clinical course of food protein-induced allergic proctocolitis: 10-year experience of a tertiary medical center. J. Allergy Clin. Immunol. Pract..

[12] Xanthakos S.A., Schwimmer J.B., Melin-Aldana H., Rothenberg M.E., Witte D.P., Cohen M.B. (2005). Prevalence and outcome of allergic colitis in healthy infants with rectal bleeding: A prospective cohort study. J. Pediatr. Gastroenterol. Nutr..

[13] Arvola T., Ruuska T., Keränen J., Hyöty H., Salminen S., Isolauri E. (2006). Rectal bleeding in infancy: Clinical, allergological, and microbiological examination. Pediatrics.

[14] Elizur A., Cohen M., Goldberg M.R., Rajuan N., Cohen A., Leshno M., Katz Y. (2012). Cow’s milk associated rectal bleeding: A population based prospective study. Pediatr. Allergy Immunol..

[15] Martin V.M., Virkud Y.V., Seay H., Hickey A., Ndahayo R., Rosow R., Southwick C., Elkort M., Gupta B., Kramer E. (2020). Prospective assessment of pediatrician-diagnosed food protein-induced allergic proctocolitis by gross or occult blood. J. Allergy Clin. Immunol. Pract..

[16] Buyuktiryaki B., Kulhas Celik I., Erdem S.B., Capanoglu M., Civelek E., Guc B.U., Guvenir H., Cakir M., Dibek Misirlioglu E., Akcal O. (2020). Risk factors influencing tolerance and clinical features of food protein-induced allergic proctocolitis. J. Pediatr. Gastroenterol. Nutr..

[17] Koksal B.T., Barış Z., Ozcay F., Yilmaz Ozbek O. (2018). Single and multiple food allergies in infants with proctocolitis. Allergol. Immunopathol..

[18] Celik P., Yilmaz D., Yuksel F., Akpinar F., Karabag K., Uzun A.K., Dibek Misirlioglu E. (2025). Behavioral feeding problems and parenting stress in toddlers with food protein-induced allergic proctocolitis. Ann. Allergy Asthma Immunol..

[19] Concha S., Cabalín C., Iturriaga C., Pérez-Mateluna G., Gomez C., Cifuentes L., Harris P.R., Gana J.C., Borzutzky A. (2018). Diagnostic validity of fecal occult blood test in infants with food protein-induced allergic proctocolitis. Rev. Chil. Pediatr..

[20] Czaja-Bulsa G., Bulsa K., Łokieć M., Drozd A. (2024). Can faecal zonulin and calprotectin levels be used in the diagnosis and follow-up in infants with milk protein-induced allergic proctocolitis?. Nutrients.

[21] Lake A.M. (2000). Food-induced eosinophilic proctocolitis. J. Pediatr. Gastroenterol. Nutr..

[22] Lamont R.F., Møller Luef B., Stener Jørgensen J. (2020). Childhood inflammatory and metabolic disease following exposure to antibiotics in pregnancy, antenatally, intrapartum and neonatally. F1000Research.

[23] Huang K.Y., Liang B.S., Zhang X.Y., Chen H., Ma N., Lan J.L., Li D.Y., Zhou Z.W., Yang M. (2023). Molecular characterization of Clostridium perfringens isolates from a tertiary Children’s Hospital in Guangzhou, China, establishing an association between bacterial colonization and food allergies in infants. Gut Pathog..

[24] Nowak-Węgrzyn A. (2015). Food protein-induced enterocolitis syndrome and allergic proctocolitis. Allergy Asthma Proc..

[25] Nowak-Węgrzyn A., Katz Y., Mehr S.S., Koletzko S. (2015). Non-IgE-mediated gastrointestinal food allergy. J. Allergy Clin. Immunol..

[26] Rojas Gallegos M.B., Crissinger K.D. (2020). Outcomes of Infants with Severe Refractory Food Protein-Induced Allergic Proctocolitis Treated with Mesalamine. JPGN Rep..

[27] Chinese Medical Association of Allergy and Immunology (2022). Expert consensus on mechanisms and targeted therapy of Type 2 inflammatory diseases. Chin. Med. J..

[28] Liu W., Ma X., Zhou W. (2019). Adverse events of Benralizumab in moderate to severe eosinophilic asthma: A meta-analysis. Medicine.

[29] Chung H.L., Hwang J.B., Park J.J., Kim S.G. (2002). Expression of transforming growth factor beta1, transforming growth factor type I and II receptors, and TNF-alpha in the mucosa of the small intestine in infants with food protein-induced enterocolitis syndrome. J. Allergy Clin. Immunol..

[30] Morita H., Nomura I., Orihara K., Yoshida K., Akasawa A., Tachimoto H., Ohtsuka Y., Namai Y., Futamura M., Shoda T. (2013). Antigen-specific T-cell responses in patients with non-IgE-mediated gastrointestinal food allergy are predominantly skewed to T(H)2. J. Allergy Clin. Immunol..

[31] Erdem S.B., Nacaroglu H.T., Karaman S., Erdur C.B., Karkıner C.U., Can D. (2017). Tolerance development in food protein-induced allergic proctocolitis: Single centre experience. Allergol. Immunopathol..

